# Simulated Microgravity Disrupts Nuclear Factor κB Signaling and Impairs Murine Dendritic Cell Phenotype and Function

**DOI:** 10.3390/ijms24021720

**Published:** 2023-01-15

**Authors:** Gaetano Calcagno, Jeremy Jeandel, Jean-Pol Frippiat, Sandra Kaminski

**Affiliations:** Stress, Immunity, Pathogens Laboratory, SIMPA, Université de Lorraine, F-54000 Nancy, France

**Keywords:** dendritic cells, stress, gravity changes

## Abstract

During spaceflights, astronauts face different forms of stress (e.g., socio-environmental and gravity stresses) that impact physiological functions and particularly the immune system. In this context, little is known about the effect of such stress on dendritic cells (DCs). First, we showed that hypergravity, but not chronic ultra-mild stress, a socio-environmental stress, induced a less mature phenotype characterized by a decreased expression of MHCII and co-stimulatory molecules. Next, using the random positioning machine (RPM), we studied the direct effects of simulated microgravity on either splenic DCs or Flt-3L-differentiated bone marrow dendritic cells (BMDCs). Simulated microgravity was found to reduce the BM-conventional DC (cDC) and splenic cDC activation/maturation phenotype. Consistent with this, BMDCs displayed a decreased production of pro-inflammatory cytokines when exposed to microgravity compared to the normogravity condition. The induction of a more immature phenotype in microgravity than in control DCs correlated with an alteration of the NFκB signaling pathway. Since the DC phenotype is closely linked to their function, we studied the effects of microgravity on DCs and found that microgravity impaired their ability to induce naïve CD4 T cell survival, proliferation, and polarization. Thus, a deregulation of DC function is likely to induce immune deregulation, which could explain the reduced efficiency of astronauts’ immune response.

## 1. Introduction

During space missions, socio-environmental stresses and gravity changes affect many biological functions of astronauts, including the immune system. It has been shown that half of the astronauts who have spent six months on the International Space Station are prone to skin rashes, infections, and respiratory disorders such as rhinitis [1]. Numerous studies suggest that these alterations of the immune system could be explained by a lower efficiency of adaptive immunity. Indeed, mice sent into space exhibited an atrophy of lymphoid organs (spleen, thymus) linked to a lower number of lymphocytes [2]. Innate immunity also appears to be affected by gravity changes, as suggested by studies showing that spaceflights disrupt neutrophil and monocyte functions [3]. In addition, some bacteria have increased pathogenicity and may become resistant to certain antibiotics during spaceflight [4]. Furthermore, latent viruses such as herpes, varicella, or cytomegalovirus reactivate in most astronauts while in space [5,6]. These findings highlight the risks of pathology development during space missions.

Dendritic cells (DCs) are a heterogeneous cell population that, in mice, can be divided, into two cell types: conventional DCs (cDCs) and plasmacytoid DCs (pDCs) specialized in the antiviral response. cDCs and pDCs are derived from a common precursor and their development is dependent on fms-like tyrosine kinase 3 ligand (Flt-3L) [7,8]. GM-CSF (Granulocyte-Macrophage Colony Stimulating Factor) is another important cytokine for DC development and has been widely used to differentiate DCs from bone marrow in vitro [9]. Obtaining BMDCs (Bone Marrow-derived Dendritic Cells) in vitro by treating the cells with GM-CSF leads to the acquisition of an immunogenic phenotype [10]. Conversely, differentiation of BMDCs with Flt-3L produces both cDCs and pDCs with a phenotype close to their in vivo steady state [11]. Both cDC and pDC populations are of innate immunity and can orchestrate the adaptive immune response through the presentation of antigens and the secretion of cytokines to induce T cells activation. Immature mouse DCs show low levels of membrane expression of various co-stimulatory molecules, such as CD80, CD86, and CD40, and of the Major Histocompatibility Complex class II (MHCII) [12]. In addition, they exhibit low migratory capabilities and high antigen capture and priming capacity [13]. Through their membrane receptors of the PRR (Pattern Recognition Receptors) family, such as TLR (Toll-Like Receptor), they are able to recognize danger signals, which will activate different signaling pathways (ERK1/2, JNK, p38 MAPK, NFκB) that promote DC survival and activation/maturation. Upon activation/maturation, DCs acquire the ability to present antigen. This stage is characterized by an increase in the expression of MHCII and co-stimulatory molecules on their surface, a decrease in antigen priming capacity, and a rearrangement of adhesion molecules, allowing their migration [12]. Once the antigen is exposed on the DC surface, it migrates to the lymph nodes for presentation to naive CD4 T cells. Depending on the presented antigen and the produced cytokines by DCs, naïve CD4 T cell polarization will either be directed toward immunogenic (e.g., Th1, Th2 or Th17) or tolerogenic (regulatory T cell: Treg) profiles [14]. Interestingly, immature DCs with low expression of MHCII and co-stimulatory molecules (CD80, CD86, CD40) and low production of pro-inflammatory cytokines (IL-6, IL-1β, IP-10) have been shown to induce a tolerogenic response [15]. Furthermore, tolerogenic DCs can be characterized by the production of anti-inflammatory cytokines (e.g., IL-10) and the expression of tolerance markers (e.g., PDL1A, CTLA4) [16]. Thus, DCs play an essential role in maintaining immune system homeostasis, as well as that of the body, by regulating the balance between immunogenic and tolerogenic factors. 

Although DCs are at the center of the immune response, few studies have investigated the impact of spaceflight stresses on DCs. While studies on acute and chronic socio-environmental stresses showed that DCs are mainly modulated by stress hormones (e.g., corticosterone and catecholamine) [17,18], data on the influence of gravity on DCs remain incomplete. Indeed, it has been demonstrated that gravity variations impact DCs’ differentiation, phagocytosis, cytokine production, survival, and immunogenicity [19,20,21,22,23]. Human pDCs and cDCs development has been shown to be decreased under simulated microgravity conditions (21 days) [19]. Furthermore, Tackett and colleagues showed that under short-term simulated microgravity (<72 h), JAWS II DCs from a murine cell line displayed an increased expression of MHCII and CD80, as well as IL-6 production associated with an increased ability to activate both CD4 and CD8 T cells [23]. Similar results were obtained in GM-CSF BMDCs with increased expression of MHCI, CD86, and CD40. Conversely, long-term exposure to microgravity (4–14 days) has the opposite effect [23]. Another study showed that the maturation phenotype of human DCs was affected by simulated microgravity [24].

Given the lack of knowledge on the effect of spaceflight stresses on DCs, we questioned whether DCs were sensitive to different kinds of stresses encountered during spaceflights, such as socio-environmental and gravity stresses. To investigate this, DCs were incubated with serum from mice exposed to different types of stress or exposed directly to simulated microgravity using the random positioning machine (RPM). Furthermore, we used either murine splenic DCs or Flt-3L-differentiated BMDCs, which display a phenotype close to the DCs’ in vivo steady state and allowed studying cDCs and pDCs. Our data revealed that gravity affects the DC maturation/activation phenotype only in the cDC population. These phenotype alterations lead to an impairment of DCs’ capacity to activate naïve CD4 T cells.

## 2. Results

### 2.1. Mechanical Stresses Affect Dendritic Cell Phenotype

**Figure 1:**
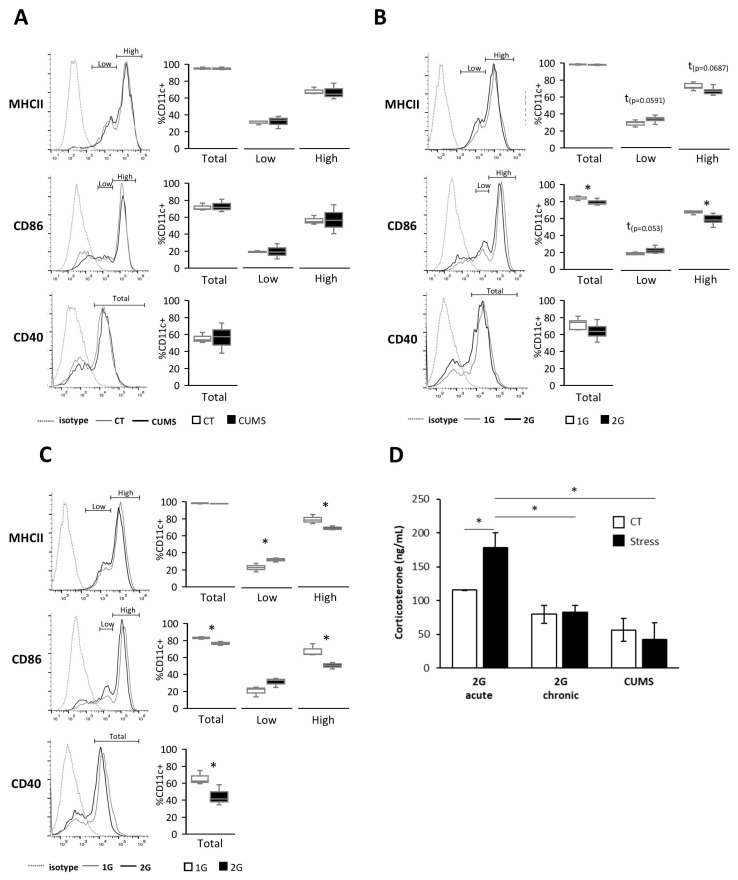
Acute and chronic hypergravity stresses affect the splenic DC phenotype independently of glucocorticoid level. (**A**–**C**) Splenic DCs were isolated from mice and cultured for 24h with serum from control mice (CT) or mice exposed to CUMS (CUMS) (**A**), chronic (**B**), or acute hypergravity (**C**) stresses. Cells were then analyzed by flow cytometry for the expression of MHCII, CD86, and CD40. Representative FACS histograms are shown of populations that express either total or low/high levels of the related marker (left panel), and box plots are shown of the median percent of CD11C gated cells expressing MHCII, CD86, or CD40 (right panel). The boxes extend from the 25th to the 75th percentile; whiskers indicate the minimum and maximum. Data are from one experiment with *n* = 3 serums/group (**A**), three independent experiments with *n* = 3 serums/group/experiment (**B**), and two independent experiments with *n* = 3 serums/group/experiment (**C**). (**D**) Mean ± SEM of corticosterone levels measured by ELISA in serum from mice submitted to chronic (*n* = 8) or acute (*n* = 3) hypergravity or CUMS (*n* = 3) stresses. *t*-test, Mann–Whitney, or ANOVA tests were used to reveal statistically significant differences. * *p* < 0.05, t indicates a tendency.

To evaluate the impact of different types of stress on the DC activation/maturation phenotype, splenic DCs were isolated and cultured during 24 h with serum from mice exposed to either mechanical (acute (3 h) or chronic (21 days)) hypergravity or socio-environmental (CUMS) stresses. Cells were then analyzed for the expression of MHCII and the co-stimulatory molecules CD40 and CD86 using flow cytometry. Since two populations of cells were distinguishable for the expression of MHCII and CD86 at high or low levels, positive populations were separated following the expression level of the respective marker. We observed that CUMS did not affect the DC phenotype (Figure 1A), in contrast to acute hypergravity stress and, to a lesser extent, chronic hypergravity stress (Figure 1B,C). Indeed, DCs exposed to serum from mice subjected to 3 h of hypergravity expressed lower levels of MHCII, CD86, and CD40 than their 1G counterparts (Figure 1B). Twenty-one days of hypergravity resulted in a slightly decreased expression of MHCII (tendency *p* = 0.0687) and CD86, while no change was detected in CD40 expression (Figure 1C). Thus, these results showed that hypergravity impairs the DC activation/maturation phenotype.

Because stress hormone secretion, such as that of corticosterone, is known to inhibit immune cells and to compromise DC maturation [18], we analyzed corticosterone’s concentration in mice serums. As shown Figure 1D, only acute hypergravity-stressed mice showed increased levels of corticosterone compared to control mice. 

Together, these results suggest that corticosterone may not be responsible for the decreased DC activation/maturation phenotype observed in DCs treated with serums issued from mice submitted to acute or chronic hypergravity stress.

### 2.2. Simulated Microgravity Impairs cDC but Not pDC Activation/Maturation Phenotype

Since many factors present in the serum from mice submitted to hypergravity could be responsible for the decreased DC maturation phenotype, we used the RPM, a system that allows the study of the gravity changes on DCs in the absence of any influence of the serum environment. To do so, BMDCs differentiated for 7 days with Flt3L or splenic DCs were cultured for 24 h in simulated microgravity and activated with LPS. The same parameters from the previous experiment were studied, as well as the co-stimulatory molecule CD80. 

**Figure 2:**
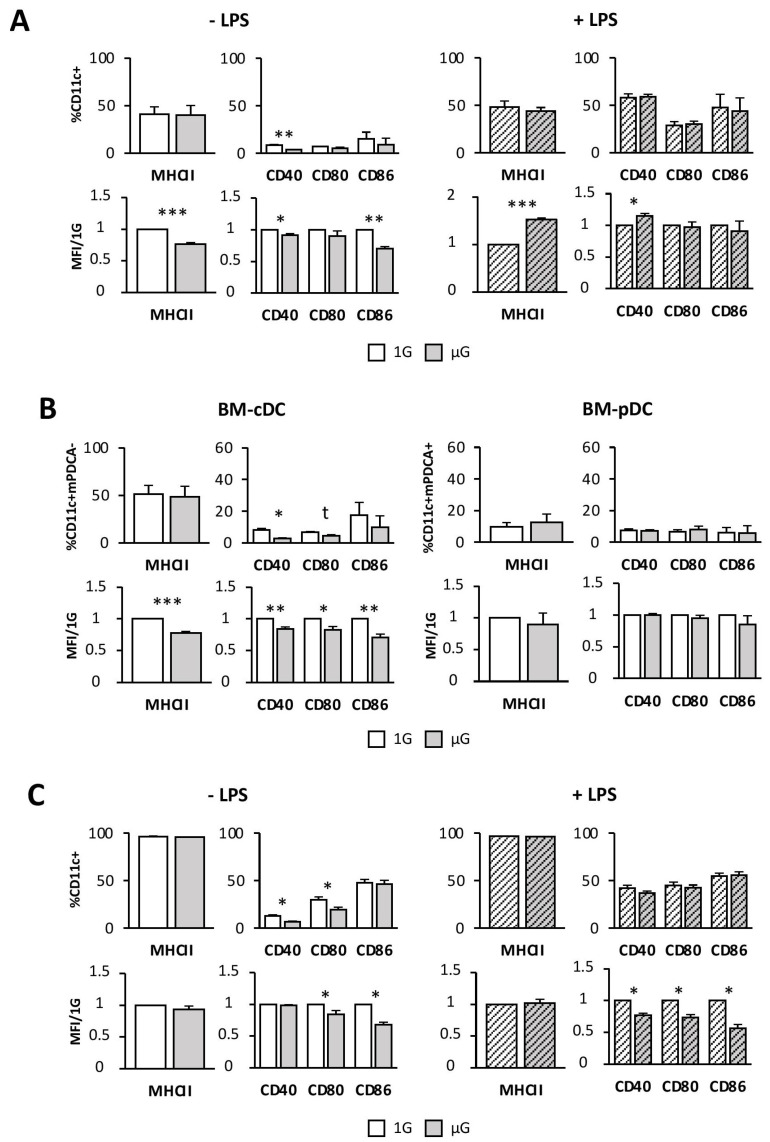
Simulated microgravity alters cDCs but not pDC phenotype. BMDCs differentiated for 7 days with Flt3L (**A**,**B**) or splenic DC (**C**) were cultured for 24 h in the absence (1G) or presence (µG) of simulated microgravity and with (+LPS) or without (−LPS) LPS. Cells were then analyzed by flow cytometry for the expression levels of MHCII, CD40, CD80, and CD86 in CD11C+, CD11C+ mPDCA− (cDC) or CD11C+ mPDCA+ (pDC) populations. (**A**–**C**) Mean percent ± SEM of gated cells expressing MHCII, CD40, CD80, and CD86 (top panel). Mean MFI ± SEM with 1G values set as 1. Data were normalized and are expressed as fold differences relative to 1G cells (bottom panel). Data are from three (**A**,**B**) or four (**C**) independent experiments. *t*-test or Mann–Whitney test was used to reveal statistically significant differences. * *p* < 0.05, ** *p* < 0.01; *** *p* < 0.001, t indicates a tendency.

We first showed that, after microgravity exposure, Flt3L-differentiated BMDCs expressed lower levels of the different markers, compared to the 1G condition, consistent with the in vivo studies (Figure 2A and Figure S1A). Thus, we found that microgravity affected CD40 expression, not only as the percentage of cells expressing CD40 (3.8% in µG vs. 8.4% in 1G) (Figure 1A upper panel and Figure S1A), but also with respect to intensity of expression per cell (8% less than control) (MFI, Figure 1A lower panel and Figure S1A), as compared to 1G BMDCs. While no difference was found for the proportion of inactivated cells expressing MHCII and CD86, their MFI ratios were lower in BMDCs submitted to microgravity than in 1G BMDCs (23% decrease for MHCII and 30% for CD86) (Figure 1A lower panel and Figure S1A). After activation with LPS, BMDCs presented a phenotype comparable to that of their 1G counterpart, with an even higher level of MHCII and CD40 expression intensity (Figure 1A lower right panel and Figure S1A). 

These results led us to characterize BM-cDCs and BM-pDCs in more detail. BM-cDCs subjected to simulated microgravity displayed a more immature phenotype than that of 1G BM-cDCs, as indicated by the lower percentage of cells expressing CD40 (2.6% in µG compared to 8% in 1G) and CD80 (4.5% in µG vs. 6.6% in 1G) (Figure 2B left upper panel and Figure S1B) and by the lower MFI ratios of all studied markers (decreases of 22%, 17%, 17% and 29% for MHCII, CD40, CD80, and CD86, respectively) (Figure 2B left lower panel and Figure S1B). No phenotype changes following microgravity were observed in BM-pDCs (Figure 2B right panel and Figure S1B). Thus, these results showed that BM-cDCs, but not BM-pDCs, were sensitive to microgravity.

To confirm the impact of simulated microgravity on cDCs, we then repeated these experiments using splenic cDCs. Simulated microgravity altered the percentage of cells expressing the co-stimulatory molecules CD40 (6.6% for µG vs. 12.8% for 1G) and CD80 (19.5% in µG vs. 29.8% in 1G) (Figure 2C and Figure S1C), as well as the intensity of CD80 and CD86 expression (decreases of 16% and 32%, respectively), compared to control cells. Upon activation, the percentage of splenic DCs expressing MHCII and the co-stimulatory molecules reached expression equivalent to that of 1G cells. However, cell activation with LPS failed to restore the expression levels of co-stimulatory molecules to control levels (decreases of 23%, 27% and 43% for CD40, CD80, and CD86, respectively), as indicated by MFI ratios (Figure 2C and Figure S1C). These results confirm that microgravity interfered with the DC activation/maturation phenotype and that, particularly for splenic DCs, this immature phenotype was maintained despite cell activation. 

Altogether, our results demonstrated that microgravity rendered Flt3L-differentiated BMDCs and splenic DCs less mature than cells cultured upon the 1G condition and that cDC was the microgravity-sensitive DC population.

### 2.3. Simulated Microgravity Reduces Proinflammatory Cytokine Production but Does Not Induce Expression of Tolerogenic Markers

**Figure 3:**
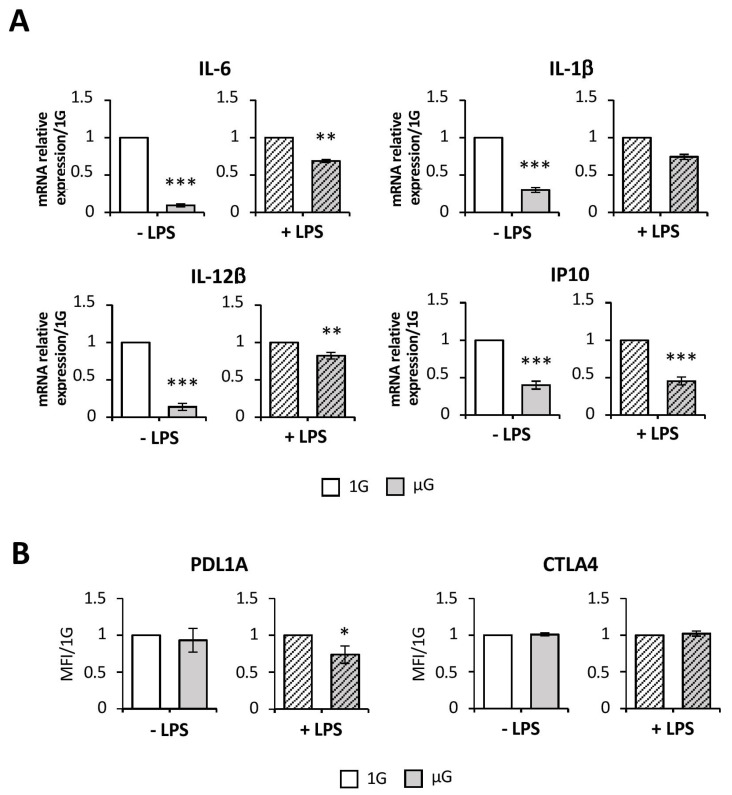
Simulated microgravity impairs pro-inflammatory cytokine production but does not increase tolerance makers in Flt3-L-differentiated BMDC. Mouse BMDCs were differentiated for 7 days with Flt3-L followed by 24 h in the absence (1G) or presence (µG) of simulated microgravity with (+LPS) or without (−LPS) LPS. (**A**) mRNA quantification of the indicated genes was performed by real-time PCR. Wild-type values were normalized to a value of 1. (**B**) Cells were analyzed by flow cytometry for the expression of PDL1A and CTLA4 in CD11C+ populations. Mean MFI ± SEM with 1G values were normalized to a value of 1. Data from three (**A**) or four (**B**) independent experiments were normalized and are expressed as fold differences relative to 1G cells. *t*-test or Mann–Whitney test was used to reveal statistically significant differences. * *p* < 0.05; ** *p* < 0.01; *** *p* < 0.001.

We then quantified IL-6, IL-1β, IL-12β, and IP-10 pro-inflammatory cytokine transcripts. As shown in Figure 3A, microgravity interfered with all tested cytokine production in both non-activated and LPS-activated BMDCs, compared to 1G cells, except for IL-1β transcripts that were only affected in inactivated cells. Indeed, we observed in non-activated BMDCs a decrease of 91% of IL-6, 71% of IL-1β, 87% of IL-12β, and 61% of IP-10 transcripts. In activated BMDCs, microgravity decreased by 32%, 18%, and 55% of IL-6, IL-12β, and IP-10, respectively.

Since an immature DC phenotype and a lower pro-inflammatory cytokine production could result in a tolerogenic DC profile [16], we evaluated tolerogenic marker expression using flow cytometry. We showed that neither CTLA4 nor PDL1A were upregulated under a simulated microgravity condition (Figure 3B) in BMDCs, compared to control cells. PDLA1 expression was even lower in LPS-activated BMDCs (Figure 3B) subjected to microgravity than in 1G BMDCs. 

Taken together, these results clearly demonstrated that simulated microgravity altered DCs’ ability to activate/maturate through the downregulation of proinflammatory cytokine expression, but without inducing tolerogenic marker expression.

### 2.4. Simulated Microgravity Could Impaired BMDC Maturation through an Alteration of NFκB Signaling Pathway

**Figure 4:**
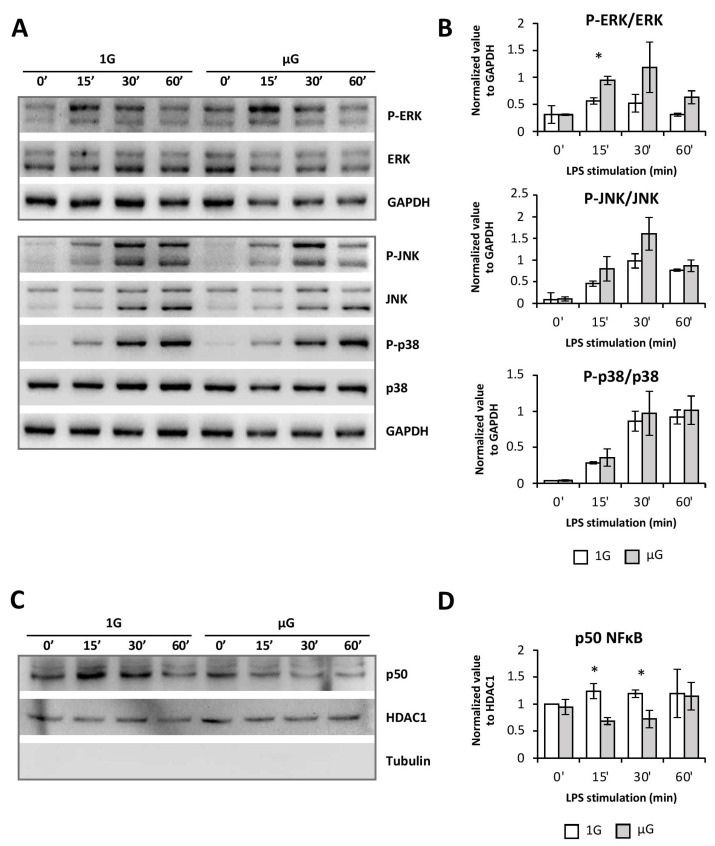
Simulated microgravity reduces NFκB signaling following BMDC activation. Mouse BMDCs were differentiated for 7 days with Flt3-L followed by 24 h in the absence (1G) or presence (µG) of simulated microgravity. Cells were then stimulated with LPS (100ng/mL) for the indicated times. (**A**,**B**) Total and phosphorylated MAPK family members in BMDCs detected by Western blot of whole-cell lysate. A representative blot out of three independent experiments with similar results is shown (**A**). Graphs represent the mean ± SEM of normalized phosphorylated/total amount ratio of respective proteins (**B**). (**C**,**D**) Nuclear extracts were used to determine the amount of translocated p50 using Western blot. A representative blot out of four independent experiments with similar results is shown (**C**). Graph represents the mean ± SEM of p50 normalized nuclear amounts in BMDC. Tubulin was used to verify the absence of cytoplasm proteins in nuclear extracts. *t*-test or Mann–Whitney test was used to reveal statistically significant differences. * *p* < 0.05.

In order to understand how simulated microgravity downregulates DC maturation markers, we next studied different signaling pathways that have been described to play a role in DC maturation and to be disturbed by gravity changes [25,26,27]. For this, Flt3L-differenciated BMDCs were submitted to simulated microgravity for 24h and then activated with LPS for 15 to 60 min. We first studied the MAPK pathway by measuring ERK1/2, JNK, and p38 phosphorylation in whole-cell extract by Western blot. Interestingly, none of these pathways was downregulated following microgravity exposure (Figure 4A,B). ERK1/2 phosphorylation was even found to be higher after 15 min of LPS activation in µG, compared to 1G BMDCs. 

We then assessed the effect of microgravity on the NFκB signaling pathway. Indeed, NFκB has been described for its essential role in the acquisition of the DC matured phenotype and particularly in the regulation of CD80 [26] and pro-inflammatory cytokine expression, such as IL-6 and IL-12 [26]. Furthermore, the NFκB signaling pathway has been shown to be dysregulated by gravity modifications in different cell types, such as T cells and macrophages [28,29]. To study this pathway, we used the same approach as was used for the MAPK pathway, but we followed p50 nuclear translocation as a marker of its activation. As shown Figure 4C,D, p50 translocation was diminished in BMDCs exposed to microgravity after 15 and 30 min compared to 1G cells.

These results showed that NFκB was impaired by microgravity, suggesting that these alterations could account for the BMDC immature profile observed in microgravity.

### 2.5. Simulated Microgravity Alters DC Function to Induce Naïve CD4 T Cell Survival, Proliferation and Polarization

**Figure 5:**
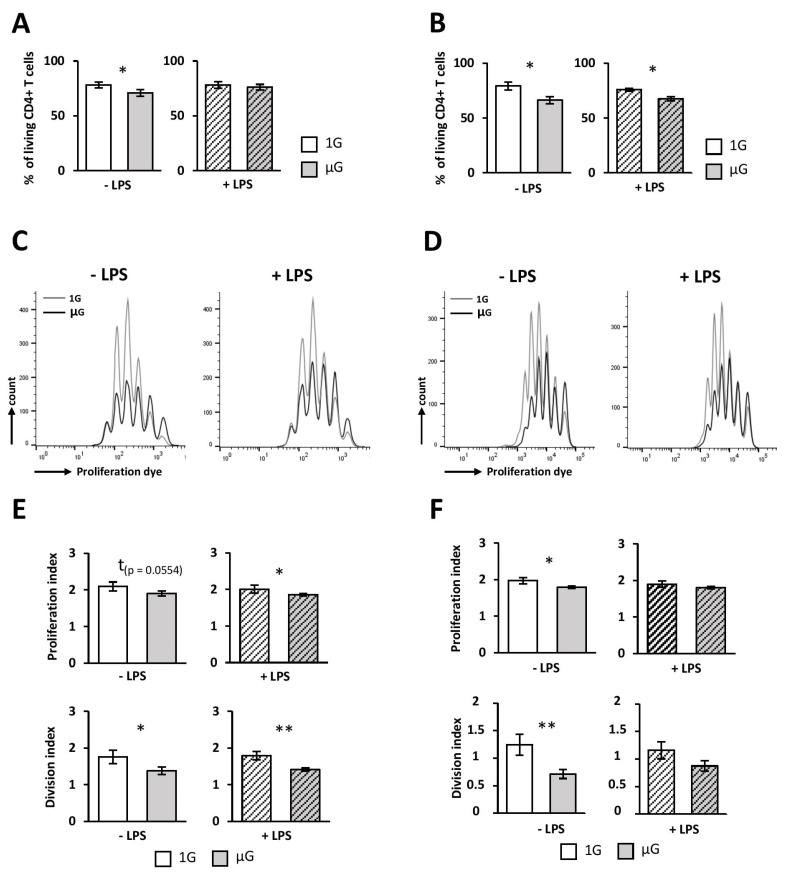
Simulated microgravity impairs DCs’ ability to stimulate CD4+ T cell proliferation and survival. Splenic DCs were cultured for 24 h in the absence (1G) or presence (µG) of simulated microgravity with (+LPS) or without (−LPS) LPS and then pulsed with ovalbumin peptide (ova_323-339_) (**A**,**C**,**E**) or full protein (**B**,**D**,**F**). DCs were then co-cultured for 3 days with mouse-isolated OTII CD4+ T cells. Cell survival (**A**,**B**) and proliferation (**C**–**F**) were assessed using flow cytometry by using dead cell or proliferation tracker, respectively. (**A**,**B**) Mean percent ± SEM of living CD4+ T cells. (**C**,**D**) Representative proliferation profile from at least 3 independent experiments with the same results. (**E**,**F**) Mean ± SEM of proliferation and division indexes calculated using FlowJo software v10.8.1 for three (**E**) or four (**F**) independent experiments. *t*-test or Mann–Whitney test was used to reveal statistically significant differences. * *p* < 0.05, ** *p* < 0.01, t indicates a tendency.

DCs need to express high levels of MHCII and CD40, CD80, and CD86 co-stimulatory molecules to mount an effective CD4 T-cell response. Indeed, productive engagement of the naïve CD4 T cell TCR leads to the delivery of signals required for their proliferation, their survival, and their differentiation into effector cells [30]. Because our results showed that simulated microgravity contributed to the emergence of a reduced pro-inflammatory profile in DCs, we next sought to determine whether simulated microgravity affects DC function. To do this, splenic DCs were exposed to simulated microgravity for 24h and stimulated with LPS. DCs were then pulsed with either ova_323-339_ peptide or full-length protein and cultivated with OTII naive CD4 T cells for 3 or 4 days to assess their proliferation and polarization, respectively. Both full-length protein and ova_323-339_ peptide were used to discriminate between a putative effect of simulated microgravity in DCs’ antigen processing/presentation processes or in their reduced inflammatory state. Survival of CD4 T cells was reduced when non-activated DCs were exposed to simulated microgravity prior to CD4 T cell activation, whether they were pulsed with ova_323-339_ (8%) or full-length protein (14%), respectively (Figure 5A,B). CD4 T-cell survival was slightly decreased when DCs were activated with LPS and pulsed with ova full-length protein, but not when pulsed with the peptide (Figure 5A,B). 

The reduced proliferation and division indices, compared to control conditions, demonstrated that CD4 T-cell proliferation was impaired when CD4 T cells were activated with DCs exposed for 24 h to microgravity, consistent with cell survival (Figure 5C–F). Indeed, CD4 T-cell proliferation and division indices were reduced by approximately 10% and 20%, respectively, when non-activated or LPS-activated ova_323-339-_pulsed DCs were used to stimulate CD4 T cells in the µG, compared to the 1G condition (Figure 5C,E). These results showed that less CD4 T cells can proliferate and those that enter into division proliferate less. Comparable results were obtained under the µG condition using full-length ovalbumin to pulse DCs with proliferation and division indices reduced by approximately 10% and 40%, respectively, compared to the 1G condition (Figure 5D,F). Under these conditions, no differences were found when LPS-activated DCs were used to stimulate naïve CD4 T cells (Figure 5D,F). 

**Figure 6:**
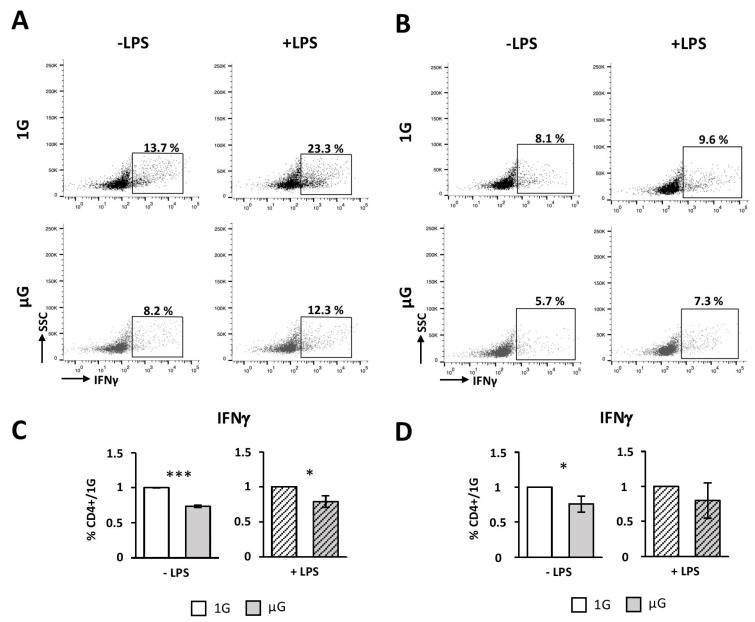
Simulated microgravity impairs the ability of DCs to polarize CD4+ T cells towards Th1 profile. Splenic DCs were isolated from mice and cultured for 24 h in the absence (1G) or presence (µG) of simulated microgravity with (+LPS) or without (−LPS) LPS and then pulsed with ovalbumin peptide (ova_323-339_) (**A**,**C**) or full protein (**B**,**D**). DCs were then co-cultured for 4 days with CD4+ T cells isolated from OTII mice. On day 4, T cells were re-stimulated for 6 h with PMA/ionomycin in the presence of Golgi inhibitor. Production of IFNγ was determined by intracellular staining followed by flow cytometry. (**A**,**B**) Representative FACS dotplots are shown from three (**A**) or four (**B**) independent experiments. Numbers in the quadrant represent the percentage of IFNγ-producing CD4+ T cells for each condition. (**C**,**D**) Mean ± SEM of 3 independent experiments, 1G values were set as 1. Data from three (**C**) or four (**D**) independent experiments were normalized and are expressed as fold differences relative to 1G cells. *t*-test or Mann–Whitney test was used to reveal statistically significant differences. * *p* < 0.05; *** *p* < 0.001.

We then investigated the ability of microgravity-exposed DCs to polarize naïve CD4 T cells through Th1, Th2, Th17, or Treg. As shown in Table S1, ova_323-339-_pulsed DCs polarized mainly naïve CD4 T cells toward Th1 effector cells characterized by the production of IFNγ. Our results revealed that both activated and non-activated DCs pulsed with ova_323-339_ showed an approximately 20–25% reduced ability to polarize naïve CD4 T cells toward Th1, compared to DCs cultivated in 1G (Figure 6A,C). Although the induction of Th1 cells was less effective, a reduced percentage of about 25% of Th1 cells was obtained when DCs were pulsed with the ovalbumin protein (Figure 6B,D), compared to their 1G counterpart. As for proliferation, no differences were found between µG and 1G conditions when LPS-activated DCs were used to stimulate naïve CD4 T cells (Figure 6B,D).

Taken together, these data demonstrate the microgravity-impaired DCs’ capacity to activate naïve CD4 T cells, leading to a reduction in their capacity to survive, to proliferate, and to polarize toward a pro-inflammatory profile. 

## 3. Discussion

Different forms of stress encountered during spaceflight are known to alter the immune system, but little is known about their effects on DCs [23,24]. In this study, we showed, in vitro, using serum from mice exposed to different stresses, that DCs are sensitive to both acute and chronic hypergravity stresses but not to CUMS, which is a form of socio-environmental stress. Indeed, only serums from mice submitted to hypergravity induced a lower expression of DC activation/maturation markers, such as MHCII, CD40, and CD86, compared to their control counterparts. Stresses are known to induce, via the HPA axis activation, the release of glucocorticoids from which corticosterone has been described to have anti-inflammatory effects on immune cells [31]. Thus, to explain our results, we measured corticosterone levels in mice serums obtained following exposure to different forms of stress. The corticosterone level was increased only in serums from mice subjected to acute hypergravity stress. These results are consistent with those showing that neither CUMS nor chronic hypergravity stresses displayed an elevated corticosterone level in mouse serum after 21 days of stress [32,33]. While this result could explain the lack of effect of serums from CUMS-stressed mice on the DC phenotype, corticosterone is most likely not responsible for the more immature DC phenotype exposed to serum from mice under chronic hypergravity stress. Nevertheless, it is possible that the increased corticosterone levels in serum from mice submitted to acute hypergravity stress could explain the greater decrease in the expression of maturation/activation markers in DCs exposed to those serums [34].

Together, these results demonstrated that the immature phenotype of splenic DCs exposed to serum from acute and chronic hypergravity stressed mice was not due to corticosterone-inducing DC inhibition. 

In order to further investigate the effects of gravity changes on DCs’ fate, we then used the RPM, a system that allows applying microgravity directly to the cell culture in absence of any influence of the in vivo environment. Thus, we tested the effects of simulated microgravity on bone marrow Flt3L-differentiated DCs and splenic DCs activated by LPS treatment. We showed that non-matured BMDCs exposed to microgravity during 24h exhibited a more immature phenotype, compared to the control BMDCs. These results correlated with an impaired ability of BMDCs to express pro-inflammatory cytokine transcripts (IL-6, IL-1β, IP10 and IL-12). We also demonstrated that microgravity affected BM-cDCs, but not BM-pDCs of the BMDC population, while it has previously been shown to impair both cDC and pDC development [19]. Thus, once differentiated, BM-pDCs appeared to be insensitive to microgravity. 

Upon activation/maturation with LPS, BMDCs displayed a phenotype comparable to the 1G control for the expression levels of CD80 and CD86 and more activated for the expression levels of MHCII and CD40. Thus, microgravity did not impair the BMDCs’ ability to upregulate the expression of their activation markers. Interestingly, in these cells, even if the cytokine production was still decreased, compared to the 1G control except for IL-1β, differences were less important than in non-stimulated BMDCs. Thus, increased production of pro-inflammatory cytokines following BMDC activation may have enabled the phenotype recovery of BMDCs exposed to microgravity. Indeed, it has been shown that IL-1β induces expression of co-stimulatory molecules in dendritic cells [35]. To analyze BM-pDCs vs. BM-cDCs we used an anti-mPDCA1 antibody that did not allow us to analyze which population from BM-cDCs or BM-pDCs displayed a more activated phenotype after microgravity exposure. Indeed, this marker expression is upregulated after cell activation in different cell types, such as cDCs [36]. Similar treatments of splenic cDCs confirmed that microgravity led to a decreased expression of the co-stimulatory molecules on their surface but without changes in MHCII expression. Interestingly, splenic cDC activation with LPS did not restore the same expression level of the co-stimulatory molecules as the DCs cultured in 1G condition. 

Together, these results demonstrate that microgravity exposure led to a more immature phenotype correlated with lower production of pro-inflammatory cytokine, two characteristics of tolerogenic DCs. The assessment of tolerogenic marker expression, however, showed that neither CTLA-4 nor PDL1A expression was upregulated under microgravity. PDL1A expression was even reduced in stimulated BMDCs. 

Although the MAP kinase p38 and JNK pathways were unchanged, those of ERK1/2 and NFκB were modified by microgravity. Interestingly, as was shown in the Jurkat T cell line, microgravity led to enhanced phosphorylation of the MAP kinase ERK1/2 and inhibition of nuclear translocation of NFκB [37]. These pathways have been described as important during the activation/maturation process of DCs and/or during spaceflight [16,25,26,27]. In addition, studies on mouse macrophages have shown an alteration of the activation of the RAS, ERK, and NFκB pathways, preventing their differentiation after exposure to both spaceflight and simulated microgravity [29]. The exacerbation of the ERK1/2 pathway activation found in BMDCs exposed to microgravity could be related to their more immature state. Indeed, it has been shown that the ERK1/2 pathway is more sensitive to activation in immaturely blocked DCs [26]. The observed decrease of the maturation phenotype, as well as the limited production of pro-inflammatory cytokines induced by microgravity in BMDCs, is therefore not related to a decrease in MAPK activation, but rather to the impaired nuclear translocation of NFκB. While the NFκB pathway seemed to be only transiently altered, these minor changes can completely modify the cell response through the dynamics of gene expression [38,39]. Moreover, since PDL1A expression is also dependent on this signaling pathway, it could also explain its lowered expression following LPS activation/maturation of BMDCs [40].

The capacity of DCs to induce either immunity or tolerance is largely determined by their activation state [41,42]. Given our results on the DC phenotype, we expected that simulated microgravity could impact their function. Indeed, naive T-cell activation is known to require three signals. Signal 1 corresponds to T-cell receptor (TCR) recognition of the antigen/MHC complex while signal 2 involves the co-stimulatory molecules. Finally, signal 3, determined by the cytokine environment, guides T-cell differentiation and proliferation. It is well established that a lack of co-stimulation following TCR activation leads to T-cell anergy and/or tolerance [43,44]. Indeed, signal 2 leads through the interaction of CD28 and its ligands CD80 and CD86 to the diminution of the TCR signal threshold and enhances cytokine production proliferation and survival [45]. Consistent with the production of inflammatory cytokines, our polarization assay demonstrated that DCs polarize mainly naïve CD4 T cells toward a Th1 profile, whether or not the DCs are activated or exposed to microgravity [46]. However, DCs exposed to microgravity were poorer activators of naïve CD4 T cells, compared to 1G cells. Indeed, we found a decreased survival, proliferation, and Th1 polarization when DCs were submitted to simulated microgravity prior to the co-culture with naïve CD4 T cells. This process seems to be independent of the internalization and priming of ovalbumin, since similar results were observed when DCs were cultured in the presence of ova_323-339_. However, we note that naïve CD4 T cell activation with ovalbumin was weaker than with ova_323-339_, as demonstrated by the lower CD4 T-cell survival, proliferation, and Th1 percentage. In agreement with this, it has been shown that the DC process of whole proteins reduces antigen presentation and T-cell activation, compared to peptide processing [47]. 

Taken together, our data therefore suggest that the less-efficient T-cells activation by microgravity-exposed DCs would be related to their immature phenotype and could be associated with a decrease in pro-inflammatory cytokines production. 

Although our results are in agreement with the study of Savary et al., which showed that simulated microgravity impaired the human DC phenotype and function, our results vary from those of Tackett et al. [23,24]. Indeed, while Tackett and colleagues demonstrated that microgravity has different effects depending on the duration of microgravity exposure (immunogenic for short time periods or tolerogenic for long time periods), we showed that short-time exposure to microgravity (24 h) led to a decrease of the DC maturation phenotype and function. Furthermore, we report that DCs respond slightly differently whether they are immature, in steady state or activated. Such dichotomy between the results of the study of Tackett et al. and our study could be explained by various experimental parameters, such as the cell population used (GMCSF-differentiated BMDCs and the JAWII cell line vs. FLT3-differentiated BMDCs and splenic cDCs), the type of stimulation (a cocktail containing IFN-γ, IL-4, and TNFα vs. LPS), and the use of beads to allow cell anchorage vs. no use of beads [23]. 

In conclusion, our results bring a better understanding of simulated microgravity effects on DCs’ fate, and complete the few existing data in this field. Indeed, we demonstrated for the first time that simulated microgravity preferentially targets cDCs and BM-cDCs. However, further investigations would be necessary to complete the understanding of the effects of microgravity on pDCs. Indeed, this population study is essential in the context of virus reactivation observed during spaceflight [5]. Using our parameters allowed us to demonstrate that the alteration of the DC maturation phenotype by microgravity ended in a less efficient activation of naïve CD4 T cells, which could be a major issue in the context of longer spaceflights, as well as in the opening of spaceflights to a larger population of astronauts.

## 4. Materials and Methods

### 4.1. Animals

C57Bl/6J and C57Bl/6 OTII male mice aged 8–14 weeks (C57BL/6-Tg(TcraTcrb)425Cbn/Crl) were purchased from Charles River (L’arbresles, France). The animals were housed in certified animal facilities of the Bioavailability–Bioactivity (Bio-DA #B54-547-24) platform or at the Faculty of Medicine (ACBS #C54-547-30). For acclimation, the mice were housed for a week in standard cages under a controlled temperature (22 °C +/− 2 °C), controlled hygrometry (50% +/− 10%), and a 12 h light-dark cycle with food and water ad libitum. Animal studies were conducted in accordance with the European Communities Council Directive (EU 2010/63) for the use and care of laboratory animals. All experimental procedures were carried out in accordance with the ethical committee (CELMEA-66). Within 5–10 min after the end of the stress procedures, the animals were anesthetized using 5% isoflurane and then put to death by cervical dislocation before tissue sampling.

### 4.2. CUMS

C57Bl/6N mice aged 8–10 weeks were exposed to 6 different types of socio-environmental mild stress: 30° cage tilt for 1, 2, or 15 h, confinement (cage of 11 cm × 8 cm × 8 cm) during 1 or 2 h, forced cohabitation for 2 h, difficult access to food during a night period of 15 h without reducing the daily food ration, circadian rhythm disturbance (15 h of permanent lighting during a night period), and 15 h of housing in a soiled cage (50 mL of water in 1 kg of litter). Stress periods lasted 1 h in the morning, 2 h in the afternoon, and 15 h at night and were separated by at least 2 h without exposure to stress to avoid habituation. After 5 days of stress, the mice were housed in normal conditions for 2 days. After 3 weeks of CUMS, the mice were anesthetized and sacrificed for biological sample collection. This CUMS protocol was performed at INSERM UMR 894 of Pitié Salpêtrière in Paris (France). Authorization was obtained from the French Ministry of Higher Education, Research, and Innovation (authorization #00966.02).

### 4.3. Chronic Hypergravity Exposure

Standard cages (36 cm × 20 cm × 14 cm) containing four 8–10-week-old C57Bl/6J male mice were placed in a large radius centrifuge [48] with a rotational speed producing a gravity vector of 2× *g*. The mice were centrifuged for 3 weeks continuously (2G group). Sufficient food and water were provided for this time period and continuous remote monitoring of the animals was ensured by infrared video. Except for the gravity level, all environmental variables were the same as those for standard housing. The control mice were housed in the same environmental conditions in a static position. At the end of the 21 days of centrifugation, the control and 2G mice were sacrificed for biological sample collection. This protocol was performed in the INSERM U1059 in St Etienne, France (authorization #04827).

### 4.4. Acute Hypergravity Exposure

C57Bl/6J male mice (7–12 weeks old) were placed in small cages on the arms of a centrifuge. The mice were centrifuged for 3 h at a speed producing a gravity vector of 2× *g*. The control mice were housed in the same conditions as those of the centrifuged mice, except that they were placed in a static position. The mice were sacrificed for biological sample collection after centrifugation (authorization #2021-008).

### 4.5. Cell Culture

The culture media composition was RPMI 1640 medium containing 10% FBS, 0.1 mM penicillin, 0.1 mM streptomycin, 10 mM HEPES, 2 mM L-glutamine, 1 mM sodium pyruvate, 0.1% non-essential amino-acids, and 2-βmercaptoethanol (Sigma Aldrich, St. Louis, MO, USA). For DC activation, lipopolysaccharide (LPS) was added, for a final concentration of 100 ng/mL. Cells were grown at 37 °C under 5% CO_2_.

### 4.6. Simulated Microgravity Exposure

Cells were maintained under simulated microgravity conditions using a Random Positioning Machine (RPM) (Yuri GmbH, Meckenbeuren, Germany). Cells were kept in suspension using a 96-well plate (Sarstedt AG & Co, Nümbrecht, Germany). The wells were fully filled to avoid air bubbles that could damage cells and avoid microgravity simulation. Then, the culture plate was sealed with a Breathe-EASIER™ membrane (Diversified Biotech, Dedham, MA, USA) allowing gas exchange and water-vapor transmission. Culture plates were placed on the center of the inner frame of the RPM. This frame rotated independently of a second frame and both frames rotated in random directions and time intervals, resulting in randomization of the gravitational vector and thus simulating microgravity (10^−3^× *g*). The average angular rotation speed was set to 60°/s. Control cells were cultivated in the same conditions but placed on the lower platform of the RPM, and thus at 1× *g* (ground conditions). RPM experiments were conducted for 24 h at 37 °C and 5% CO_2_.

### 4.7. Splenic DC Purification

Spleen was collected and dissociated enzymatically using a spleen dissociation kit (Miltenyi Biotec GmbH, Bergish Gladbach, Germany). The spleen was then mechanically dissociated using 40 μm nylon cell EASYstrainer (Greiner bio-one, Dutscher, Brumath, France). Splenic DCs were purified using a positive selection kit (CD11c Microbeads Ultrapure, Miltenyi Biotec GmbH) following the manufacturer’s instructions. Splenic DCs were then maintained in the culture medium at a concentration of 1 × 10^6^ to 2 × 10^6^ cells/mL. The splenic DCs’ purity was assessed by flow cytometry after antibody stanning, as described below, with anti-CD11c-PE-Vio^®^770 antibody (Miltenyi Biotec GmbH, Bergish Gladbach, Germany). With this method, the degree of purity was between 85% and 95%. 

### 4.8. Bone Marrow Dendritic Cells (BMDC) Differentiation

Bone marrow from C57Bl/6J mice femur and tibia was collected and clusters were dispersed using vigorous pipetting. The cells were seeded into T75 culture flasks at 2 × 10^6^ cells/mL. Differentiation into BMDCs was induced using FMS-like tyrosine kinase 3 ligand (Flt3L) ((Peprotech, Neuilly-Sur-Seine, France) or (Miltenyi Biotec GmbH, Bergish Gladbach, Germany)) at 100 ng/mL for 7 days. At days 2 and 5, half of the medium volume was removed and replaced with a fresh culture medium containing 100ng/mL of Flt3L. At day 7, the BMDCs were recovered. The differentiation percentage was evaluated by flow cytometry at day 7 using anti-CD11c-PE-Vio^®^770 antibody (Miltenyi Biotec GmbH, Bergish Gladbach, Germany), as described below. 

### 4.9. Naïve CD4+ T Cells Isolation

Mice spleen and lymph nodes were collected and dissociated with 40 μm nylon cell EASYstrainer (Greiner bio-one, Dutscher, Brumath, France). Naïve CD4+ T cells were purified using a naïve CD4+ T cell isolation kit (Miltenyi Biotec GmbH, Bergish Gladbach, Germany), following the manufacturer’s instructions. Naïve CD4+ purity was checked by flow cytometry after staining with anti-CD4-APC antibody (Miltenyi Biotec GmbH, Bergish Gladbach, Germany), and found to be between 85% and 95%.

### 4.10. Serum Corticosterone 

Serum corticosterone concentration was measured in duplicate using a commercial ELISA kit (Corticosterone Enzyme Immunoassay Kit, Arbor Assays, Euromedex, Souffelweyersheim, France) according to the manufacturer’s instructions.

### 4.11. FACS Phenotyping

To study the activation/maturation markers, the cells were stained for 20 min at 4 °C with an anti-MHCII eFluor450, an anti-CD40 APC, an anti-CD80 FITC, an anti-CD86 PE, an anti-mPDCA PEeFluor610, and anti-CD11c PC7 antibodies. All antibodies were purchased from eBioscience, San Diego, CA, USA, and each was diluted in PBS containing 0.5% of BSA (bovine serum albumin, Sigma-Aldrich, St. Louis, MO, USA), 2 mM EDTA (Fisher Chemical, Leicerstershire, UK), and 1/20 FcR Bloking Reagent (Miltenyi Biotec GmbH, Bergish Gladbach, Germany). In steady state, pDCs and cDCs were distinguished through the expression of CD11c+mPDCA+ for pDCs and CD11c+mPDCA− cells for cDCs. To study tolerogenic markers, the same staining protocol was performed using anti-PDL1 PE (Biolegend, San Diego, CA, USA), anti-CTLA4 APC (Miltenyi Biotec GmbH, Bergish Gladbach, Germany), and anti-CD11c PC7 (eBioscience, San Diego, CA, USA) antibodies.

### 4.12. Quantitative RT-PCR

Total RNA was extracted from splenic DCs or BMDCs using the NucleoSpin^®^RNA Plus kit (Macherey-Nagel, Düren, Germany) and reverse transcribed using random primers, dNTP, RNAseout, DTT, and MML-V reverse transcriptase (all from Invitrogen, Cergy Pontoise, France), following the manufacturer’s instructions. 

qPCRs were performed in triplicate using Takyon No ROX SYBR MasterMix blue dTTP (Eurogentec, Liège, Belgium) and a Mastercycler Realplex^2^ Real-Time PCR System (Eppendorf, Hamburg, Germany). The cycling program was 5 min at 95 °C followed by 40 cycles of 15 s at 95 °C and 45 s at the annealing temperature indicated in Table 1. The relative expression of transcripts of interest was standardized using 2 housekeeping transcripts (*Gusb/Eif3f* for BMDCs and *Gusb/Ef1a* for splenic DCs) using a method previously described [32]. Primers (Eurogentec, Liège, Belgium) used to amplify transcripts were designed in different exons to avoid the amplification of potential genomic DNA traces. Primer specificity was checked using a Basic Local Alignment Search Tool (BLAST) search through the US National Center for Biotechnology Information (Bethesda, MD, USA).

### 4.13. Naïve CD4 T Cell Proliferation Assay

Splenic DCs were purified and maintained during 20 h at 1.5 × 10^6^ cells/mL in the absence or presence of microgravity (RPM, Section 4.6), with or without 5 µg/mL ovalbumin peptide (ova_323-339_, Sigma Aldrich, St. Louis, MO, USA) or 200 µg/mL ovalbumin protein (Invitrogen, Waltham, MA, USA) and LPS at 100 ng/mL. Then, OTII naïve CD4+ T cells purified from OTII mice were stained with cell-proliferation dye (eBioscience, San Diego, CA, USA), following the manufacturer’s instructions. DCs were washed with culture medium to eliminate the LPS and co-cultivated in a 96-well- plate with stained naïve OTII CD4+ T cells with a ratio of 1 DC to 5 T cells. The experiment was performed in duplicate. After 3 days of coculture, the cells were washed and stained with an anti-CD4 APC (Miltenyi Biotec GmbH, Bergish Gladbach, Germany) and a Fixable Viability Dye eFluor780 (eBioscience, San Diego, CA, USA) for 20 min at 4°C. After washing, the cells were fixed for 20 min at 4 °C with PFA 2% and then washed before analysis by flow cytometry, as described below.

### 4.14. Naïve CD4+ T Cell Polarization Assay

Purified DCs and OTII naïve CD4+ T cells were used for the proliferation assay. DCs were washed in culture medium and co-cultivated with unstained T cells with the same ratio in triplicate. After 4 days of co-culture, the cells were treated with Golgi Plug (BD Bioscience BD Pharmingen, Allschwil, Switzerland), following the manufacturer’s instructions, 20 ng/mL PMA (Phorbol Myristate Acetate), and 1 µg/mL ionomycin, both purchased from Sigma Aldrich, St. Louis, MO, USA. The cells were incubated 5 h under standard culture conditions. The cells were washed and stained with anti-CD4 APC (Miltenyi Biotec GmbH, Bergish Gladbach, Germany) and Fixable Viability Dye eFluor780 (eBioscience, San Diego, CA, USA) during 20 min at 4 °C. After a washing step, the cells were fixed and permeabilized for 30 min at 4 °C using the Fix/Perm buffer provided in the FoxP3/transcription factor staining buffer set (eBioscience, San Diego, CA, USA). After 2 washes with perm buffer (also provided in the kit), the cells were stained during 30 min at room temperature with anti-FoxP3 PE (Miltenyi Biotec GmbH, Bergish Gladbach, Germany), anti- IFNγ eFluor450 (eBioscience, San Diego, CA, USA), anti-IL4 PEcy7 (eBioscience, San Diego, CA, USA), and anti-IL17 FITC (eBioscience, San Diego, CA, USA) antibodies. The cells were then washed two times with perm buffer and analyzed by flow cytometry, as described below.

### 4.15. Flow Cytometry

Flow cytometry data were collected using the Gallios flow cytometer (Beckman Coulter, Villepinte, France) and the Celesta Sorp (BD Biosciences) from the Imaging Core Facility (PTIBC) UMS2008 IBSLor (Université de Lorraine-CNRS-INSERM). Data were analyzed using the FlowJo^®^ software v10.8.1 (BD Biosciences, Ashland, OR, USA).

### 4.16. Total and Nuclear Protein Extractions

Total protein was extracted in total buffer (10 mM HEPES pH 7.9, 0.4 mM NaCl, 1.5 mM MgCl2, 0.1 mM EGTA, 5% glycerol, 0.5% NP40) supplemented with Halt Protease and Phosphatase Inhibitor Cocktail, following the manufacturer’s instructions (Thermo Fisher, Waltham, MA, USA) at a ratio of 5 × 10^6^ cells to 30 µL of buffer. The cells were incubated in buffer for 30 min and centrifuged for 15 min at 4 °C at 13,500× *g* for 15 min at 4 °C. Supernatants containing total protein were collected. 

For the NFκB study, nuclear proteins were extracted. First, 10^7^ cells were resuspended in 150 µL of Buffer A (10 mM HEPES pH 7.9, 10 mM KCl, 0.1 mM EDTA, 0.1 mM EGTA, 1 mM DTT) supplemented with Halt Protease and Phosphatase Inhibitor Cocktail (Thermo Fisher) and incubated for 15 min on ice. NP-40 (0.6% *v*/*v*) was added to cell suspension and the cells were centrifuged at 2300× *g* for 5 min at 4 °C. Supernatant was discarded and the nucleus pellet was washed twice with Buffer A. Pellet was then incubated with shaking for 1 h at 4 °C in Buffer C (20 mM HEPES pH 7.9, 0.4 mM NaCl, 1 mM EDTA, 1 mM EGTA, 1 mM DTT) containing Halt Protease and Phosphatase Inhibitor Cocktail (Thermo Fisher). After centrifugation at 13,400× *g* for 10 min at 4 °C, the supernatant containing nuclear protein was collected. Protein extracts were quantified using the Bradford method.

### 4.17. Western Blotting

Total or nuclear extract were denatured at 95 °C for 5 min. Total and nuclear protein extracts were applied to 14% SDS-polyacrylamide gels and 10% SDS-polyacrylamide gels, respectively. Proteins were transferred to polyvinylidene difluoride (PVDF) membranes (Amersham, Buckinghamshire, UK). The membranes were incubated with primary antibodies overnight at 4 °C, then with corresponding HRP-coupled secondary antibodies for 1 h at room temperature. Blots were stripped and probed again as necessary. For loading control, an anti-GAPDH antibody or an anti-HDAC1 antibody was used for total and nuclear protein extracts, respectively. Immunodetection was performed using Pierce ECL Western blotting substrate (Thermo Fisher) and signals were visualized by chemiluminescence using a Fusion FX7 camera (Vilbert-Lourmat, Collégien, France). Signal intensity was quantified using ImageJ software v1.37 (NIH) and normalized with the loading control.

### 4.18. Statistical Analysis

Statistical analyses were performed using StatView v5.0 (SAS Institute, Cary, NC, USA) and GraphPad Prism v9 (GraphPad Holdings LLC, San Diego, CA, USA) software. For two group comparisons, Kolmogorov–Smirnov and Fisher tests were used to test for normality and variance homogeneity, respectively. If these parameters were validated, a t-test or a Mann–Whitney test was carried out. For more than two groups, normality and variance homogeneity were checked using Shapiro–Wilk and Levene tests, respectively. ANOVA tests followed by post hoc Tukey–Kramer tests for two-by-two comparisons were performed. Results were considered significant at *p* values < 0.05. Histograms are shown as the mean ± standard error of the mean (SEM). Box plots are shown as the median percent extended from the 25th to the 75th percentile and whiskers indicate the minimum and maximum.

## Figures and Tables

**Table 1:** Primers used to perform quantitative RT-PCR.

Target	Sequences	Annealing Temperature (°C)
MHCII	F 5′-TTGGCCTTTTCATCCGTCACA-3′ R 5′-GTGGATACAATAGTACCATGCT-3′	60
CD40	F 5′-GTGGTCAAGAAACCAAAGGATA-3′ R 5′-TTACCATCCTCCTGTGTGACA-3′	60
CD80	F 5′-TTTCAGACCGGGGCACATAC-3′ R 5′-ATCCTTTTAGTGTCTGCAGATG-3′	60
CD86	F 5′-GCACGGACTTGAACAACCAG-3′ R 5′-GGGCACGGCAGATATGCAG-3′	60
Gusb	F 5′-CCGATTATCCAGAGCGAGT-3′ R 5′-CTCAGCGGTGACTGGTTCG-3′	61
Eif3f	F 5′-CATCAAGGCCTATGTCAGCA-3′ R 5′-AGGTCAACTCCAATGCGTTC-3′	61
Ef1a	F 5′-AGAACCAGCCCAGAACCGAA-3′ R 5′-GCAGCTGAGACTCCTTTCCA-3′	61

## Data Availability

The data presented in this study are available on request from the corresponding author.

## Supplementary Materials

The following supporting information can be downloaded at: https://www.mdpi.com/article/10.3390/ijms24021720/s1.
