# Supplementary material for: Simulated Microgravity Disrupts Nuclear Factor κB Signaling and Impairs Murine Dendritic Cell Phenotype and Function
Source: Int J Mol Sci. 2023 Jan 15;24(2):1720. doi: 10.3390/ijms24021720 (PMC9865583; doi:10.3390/ijms24021720)
Supplement: Supplementary file 1 [file ijms-24-01720-s001.zip › ijms-2115652-supplementary.pdf]

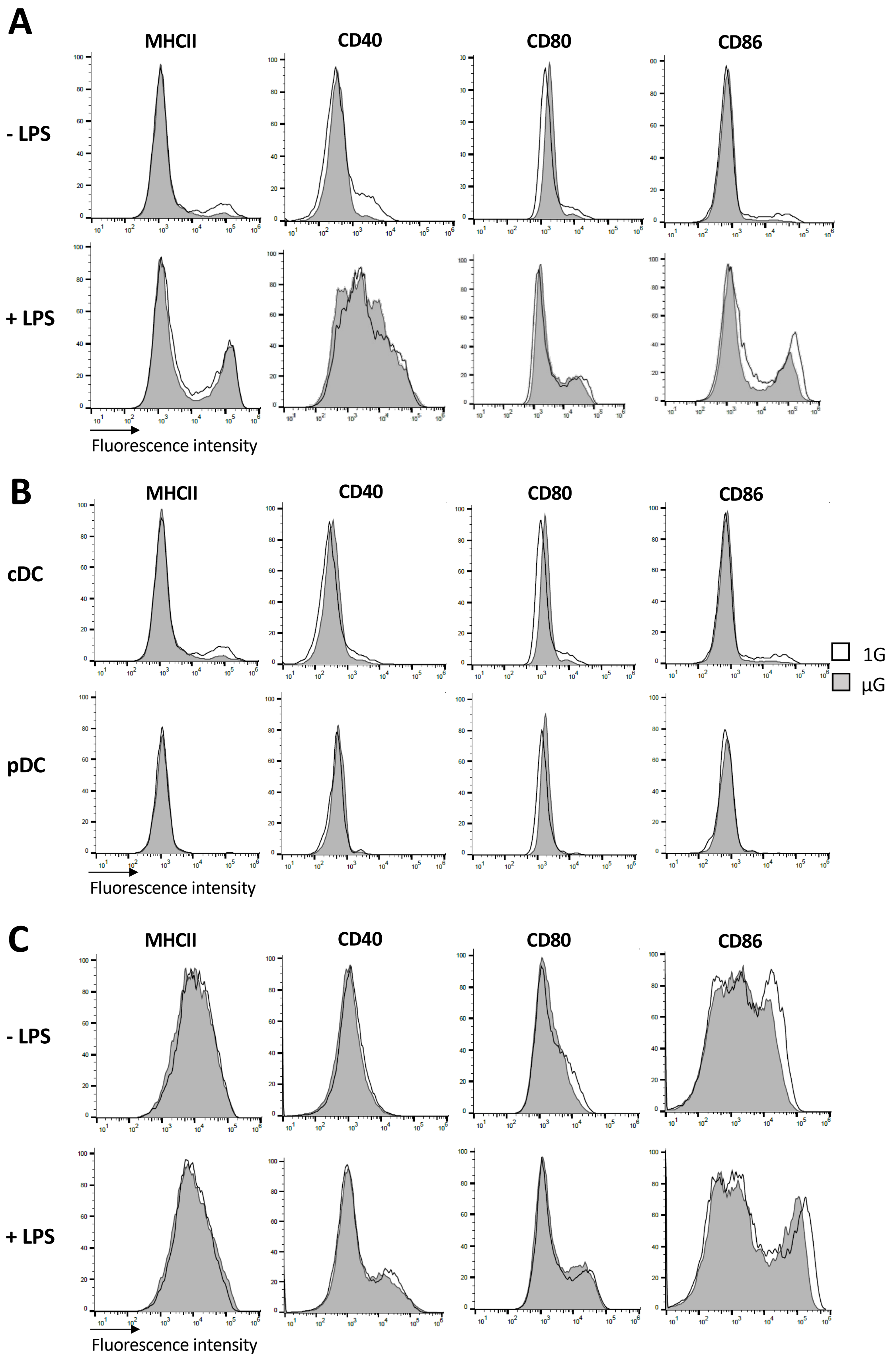

Figure S1. Simulated microgravity alters cDC but not pDC phenotype. BMDC differentiated for 7 days with Flt3L (**A-B**) or splenic DCs (**C**) were cultured for 24 hours in the absence (1G) or presence (μG) of simulated microgravity and with (+LPS) or without (-LPS) LPS. Cells were then analyzed by flow cytometry for the expression levels of MHCII, CD40, CD80 and CD86 in CD11C+, CD11C+ mPDCA- (cDC) or CD11C+ mPDCA+ (pDC) populations. (**A, B and C**) Representative FACS histograms are shown of populations that express the related marker.

Table S1. Ova323-339-pulsed DCs polarize mainly naïve CD4 T cells towards Th1 effector cells. Splenic DCs were isolated from mice and cultured for 24 hours in the absence (1G) or presence (µG) of simulated microgravity with (+LPS) or without (-LPS) LPS and then pulsed with ova323-339. DCs were then cocultured for 4 days with CD4+ T cells isolated from OTII mice. On day 4, T cells were restimulated for 6 hours with PMA/ionomycin in the presence of Golgi inhibitor. Production of indicated cytokine or transcription factor was determined by intracellular staining followed by flow cytometry. Data represent the percentage of CD4+ T cells subpopulation for each condition. Mean ± SEM of 3 independent experiments.

		**Th1 (CD4+IFNg+)**	**Th2 (CD4+ IL4+)**	**Th17 (CD4+ IL17+)**	**Treg (CD4+ FoxP3)**
**- LPS**	**1G**	13.4 +/- 2.3	2.32 +/- 1.54	0.44 +/- 0.27	0.21 +/- 0.1
	**µG**	9.83 +/- 1.66	2.46 +/-1.26	0.66 +/- 0.08	0.48 +/- 0.09
**+ LPS**	**1G**	18.29 +/- 1.55	3.18 +/- 2.61	0.22 +/- 0.07	0.13 +/- 0.05
	**µG**	14.11 +/- 0.7	1.93 +/- 1.27	0.27 +/- 0.08	0.21 +/- 0.06
